# Mitochondrial Dyshomeostasis as an Early Hallmark and a Therapeutic Target in Amyotrophic Lateral Sclerosis

**DOI:** 10.3390/ijms242316833

**Published:** 2023-11-27

**Authors:** Natalia V. Belosludtseva, Lyudmila A. Matveeva, Konstantin N. Belosludtsev

**Affiliations:** 1 Institute of Theoretical and Experimental Biophysics, Russian Academy of Sciences, Institutskaya 3, Pushchino 142290, Russia; nata.imagination@gmail.com; 2 Department of Biochemistry, Cell Biology and Microbiology, Mari State University, pl. Lenina 1, Yoshkar-Ola 424001, Russia; matveeva.lyudmilla@yandex.ru

**Keywords:** amyotrophic lateral sclerosis, SOD1, mitochondria, mitochondrial dyshomeostasis, mitophagy, mitochondrial biogenesis, mitochondrial dynamics, oxidative stress, bioenergetic stress

## Abstract

Amyotrophic lateral sclerosis (ALS) is a fatal multisystem disease characterized by progressive death of motor neurons, loss of muscle mass, and impaired energy metabolism. More than 40 genes are now known to be associated with ALS, which together account for the majority of familial forms of ALS and only 10% of sporadic ALS cases. To date, there is no consensus on the pathogenesis of ALS, which makes it difficult to develop effective therapy. Accumulating evidence indicates that mitochondria, which play an important role in cellular homeostasis, are the earliest targets in ALS, and abnormalities in their structure and functions contribute to the development of bioenergetic stress and disease progression. Mitochondria are known to be highly dynamic organelles, and their stability is maintained through a number of key regulatory pathways. Mitochondrial homeostasis is dynamically regulated via mitochondrial biogenesis, clearance, fission/fusion, and trafficking; however, the processes providing “quality control” and distribution of the organelles are prone to dysregulation in ALS. Here, we systematically summarized changes in mitochondrial turnover, dynamics, calcium homeostasis, and alterations in mitochondrial transport and functions to provide in-depth insights into disease progression pathways, which may have a significant impact on current symptomatic therapies and personalized treatment programs for patients with ALS.

## 1. Introduction

Amyotrophic lateral sclerosis (ALS) is a progressive neurodegenerative disease that leads to severe disability due to increasing muscle weakness, atrophy, spasticity and, ultimately, to paralysis of striated muscles and early death, usually within 3–5 years after the onset of symptoms [1,2,3]. ALS belongs to the group of motor neuron diseases and is characterized by steadily progressive degeneration and death of motor neurons of the cerebral cortex, brainstem and anterior horns of the spinal cord (upper and lower motor neurons, correspondingly). The death of the upper motor neurons leads to spasticity and increased excitability, while the loss of the lower motor neurons directly innervating skeletal muscles causes increasing muscular atrophy and subsequent progressive paralysis [4,5]. Impaired energy metabolism, loss of muscle and adipose tissue mass, and significant weight loss are also common and severe symptoms of ALS, caused by both an abnormally high metabolic rate in the organism and inadequate caloric intake [6,7].

Despite all the numerous efforts made, to this day ALS remains an incurable and a poorly understood disease. About 90% of cases are sporadic, while the remaining 10% of cases are familial and are mainly associated with mutations in the genes encoding Cu/Zn superoxide dismutase 1 (SOD1) and C9orf72 (chromosome 9 open reading frame 72) proteins. Studies within the last few decades have revealed more than 40 novel genes associated with ALS; a number of transgenic animal models of the disease have been developed [7,8,9]. However, the development of successful methods of treating the disease is still hampered by the lack of fundamental knowledge about how these ALS-associated mutations contribute to ALS etiology and, as a result, a limited number of therapeutic targets. According to current understanding of the pathogenesis of ALS, the degeneration and ultimately the death of a motor neuron may be due to complex mechanisms of interaction of various pathogenetic processes, such as the formation of abnormal protein aggregates, impaired RNA metabolism, neuroinflammation, defective autophagy, glutamate excitotoxicity, deprivation of neurotrophic factors, impaired axonal, exosomal and calcium transport, oxidative stress, and mitochondrial dysfunction [7,10,11,12].

Mitochondria play a crucial role in energy, ionic, and redox metabolisms, as well as the maintenance of cellular homeostasis, including the balance of protein metabolism (proteostasis) and RNA metabolism (ribostasis), which are essential for the structural integrity and functioning of all components of the neuromuscular system under pathophysiological conditions [13,14,15]. Recent studies point to the development of defects in “mitochondrial homeostasis” in ALS as a possible pathogenetic pathway that deserves a more detailed study [7,13,16]. Mitochondrial homeostasis or mitostasis refers to the maintenance of the adequate quantity and quality of the organelles and their internal environment, which is dynamically regulated by oxidative phosphorylation, mitochondrial dynamics (the fission/fusion processes), trafficking, and quality control systems (mitochondrial biogenesis and clearance) [17].

In recent years, it has become obvious that the steadily progressive death of motor neurons and the increase in myopathy in ALS are directly related to the dysregulation of mitochondrial homeostasis and bioenergetic stress. Numerous studies revealed that the accumulation of abnormal mitochondria is closely correlated with the onset of denervation in ALS patients and many in vitro and in vivo models of ALS [18,19,20]. Studies showed that several ALS-related genes, including the most well-known *SOD1* and the recently identified *SQSTM1*, *OPTN*, and *TBK1*, play a direct role in the regulation of mitochondrial function [21]. For example, the mutant protein SOD1 can accumulate on the outer membrane of mitochondria [22] and cause defects in the functioning of respiratory chain complexes [23]. It is noteworthy that ALS-linked mutations in *OPTN*, *SQSTM1*, and *TBK1* can interfere with selective degradation of mitochondria via autophagy (mitophagy) and lead to inefficient turnover of damaged mitochondria, which may contribute to the progression of the neurodegenerative disease [24,25]. Furthermore, the significance of maintaining a pool of functional mitochondria in ALS was confirmed by the data on neuroprotective effects of a number of mitochondria-targeted compounds (in particular, antioxidants MtoQ, Mito-CP, SS-31, coenzyme A, and others) [26,27,28,29,30,31,32]. Therefore, mitochondrial malfunction seems to be due to the mutation of several genes associated with ALS and plays a crucial role in the pathogenesis of the disease. However, the molecular details relating to the complex relationship between mitochondrial abnormalities and specific alterations in cells leading to neuromuscular degeneration are still poorly understood.

This review provides an update on recent progress in the study of the causes and progression of dyshomeostasis of mitochondrial processes in the context of ALS, with particular emphasis on new understanding of the role of mitochondrial quality control systems and remodeling of the mitochondrial network in the development of ALS. A comprehensive characterization of mitochondrial dysfunction in ALS-affected cells and its underlying molecular mechanisms may provide insight into novel therapeutic approaches, which may have a significant impact on current symptomatic therapies and personalized treatment programs for patients with ALS.

## 2. Etiology and Physiopathology of ALS

ALS is the third leading neurodegenerative disease worldwide with a frequency of 2–2.5 per 10 thousand people per year and a prevalence of about 5 cases per 100 thousand people. Most cases of ALS (90–95%) are sporadic (sALS), while about 10% of cases are inherited in an autosomal dominant manner and, more rarely, in an autosomal recessive or X-linked manner and belong to familial forms of ALS (fALS) [7]. Two forms of the disease—sALS and fALS—are clinically indistinguishable, since in both cases similar pathological signs develop. Respiratory muscle denervation and ventilatory failure are the cause of lethal paralysis in most cases of ALS [2,7,33].

ALS was initially referred to as multiple proteinopathies, suggesting that mutant proteins in some cases of ALS may exhibit prion-like activity and induce the formation of intracellular deposits. Indeed, excessive accumulation and aggregation of mutant proteins occur at the initial stages of the pathological process, and then normally functioning cellular proteins can be involved in this process [1,33]. This phenomenon was found to be exacerbated by changes in two major protein clearance pathways, the ubiquitin-proteasome system and autophagy [21,33]. Then, it was observed that some ALS-related proteins are regulators of the metabolism of mRNAs and noncoding RNAs (ncRNAs) [34,35], leading to a further interpretation of the disease as an RNA-mediated neuropathology, which better reflects its heterogeneity.

Currently, mutations in more than 40 genes involved in various cellular processes have been linked to ALS, including proteostasis, ribostasis, DNA repair, cytoskeletal dynamics, and trafficking [36,37,38]. Among them, mutations in four genes, namely *C9ORF72* (chromosome 9 open reading frame 72), *SOD1* (Cu/n superoxide dismutase 1), *TARDBP* (transactive response DNA-binding protein 43 kDa), and *FUS* (fused in sarcoma), account for up to 70% of fALS. Other ALS-associated mutations were found in genes encoding a variety of proteins: *ALS2*, *SETX*, *SPG1 1*, *VAPB*, *ANG*, *FIG 4*, *SIG-MA1*, *HNRNPA1*, *SQSTM1*, *VCP*, *PFN1*, *OPTN* [36,37], *TLS* [39], *UBQLN2* [40], and others [36]. Furthermore, an increasing number of studies report the presence of two or more mutations in different genes in ALS patients, suggesting the oligogenic inheritance of the disease in some cases [36]. In about 20% of fALS cases, the disease occurs due to mutations in the gene encoding the antioxidant enzyme SOD1, which in a healthy cell catalyzes the dismutation of superoxide anions into molecular oxygen and hydrogen peroxide [41,42]. Mutations in this gene were the very first to be discovered and most studied in genetic models of ALS.

Patients who do not have affected relatives are classified as having sALS [11]. In the cases of sALS, in addition to genetic mutations, environmental factors are of great importance, including smoking, toxins, heavy metals, pesticides, traumatic brain injuries, viral infections, etc. [43]. This may explain the difficulties in finding individual risk factors in ALS patients and bring the pathology closer to oncological diseases, since both diseases are apparently due to the consistent impact of many factors [11,44,45].

**Figure 1:**
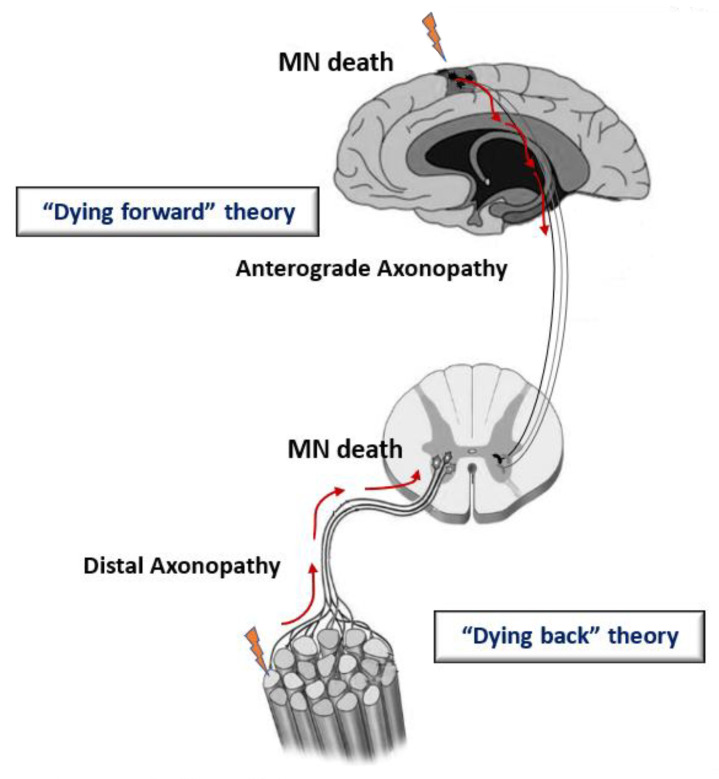
Schematic representation illustrating the theories for ALS onset. The “dying-forward” and “dying-back” hypotheses propose that pathological changes from the site of ALS origin move in the anterograde or retrograde direction, respectively, leading to the death of motor neuron (MN) cells.

At the moment, there are several hypotheses about the site of origin of ALS in the body [3,46,47]. Among them, there are “dying forward” and “dying back” hypotheses of the development of ALS—depending on which structures in the chain “upper motor neuron—lower motor neuron—skeletal muscle” are affected first (Figure 1). According to the “dying-forward” hypothesis, the pathological process begins with the upper motor neurons and moves in an anterograde direction from the central nervous system via glutamate-mediated excitotoxic processes, resulting in anterior horn cell metabolic deficit [46]. According to the “dying-back” hypothesis, the pathological process begins in the skeletal muscle and/or neuromuscular junction [48,49,50], which leads to the launch of degeneration of the lower and then the upper motor neurons [51].

Recent data suggest that the death of motor neurons is the result of pathogenetic processes occurring not only in motor neurons, but also in glial cells (astrocytes and microglia) and muscle fibers [4,52]. Moreover, studies demonstrated that ALS is not simply a disease of motor neurons, but rather a complex, multisystemic, and multifactorial disease that involves sensory neurons, interneurons, glial cells, and many other peripheral tissues [4,7,53]. Further time-resolved studies may provide important insight into the causal molecular mechanisms of cellular dysfunction, and thus represent an important tool for characterizing the pathophysiology of ALS.

## 3. Mitochondrial Malfunction and Dyshomeostasis in Patients and Genetic Models of ALS

A growing body of evidence indicates that, at the intracellular level, mitochondria are the earliest targets in ALS, and abnormalities in their complex ultrastructure, dynamics, trafficking, turnover, and functions, referred to as mitochondrial homeostasis, are contributes to the development of bioenergetic stress in ALS-affected cells and the progression of the disease. Many studies showed that mitochondrial alterations precede behavioral changes in ALS patients and animal models, suggesting that mitochondrial dyshomeostasis is an early event in the pathogenesis of the disease [13,20,30,32].

Mitochondria are known to be highly motile organelles that undergo fission and fusion, and the dynamic stability of mitochondrial tubular networks is maintained through a number of key regulatory pathways. The unique feature of mitochondrion is the presence of its own genome in the form of mitochondrial DNA (mtDNA) and a separate molecular machinery to serve mDNA and RNA maintenance and protein biosynthesis, which guarantees the functional activity of these organelles and the rest of the cell. Together with nuclear-encoded factors, mtDNA contributes to mitochondrial biogenesis, clearance, and dynamics, which provides the “quality control” of the organelles in cells [14,54,55]. Moreover, neuronal mitochondria are characterized by rapid transport along axons, in both the retrograde and anterograde directions (i.e., from distal sites to the soma and vice versa), and shifts in the rate-limiting components of the mitochondrial transport apparatus can lead to an imbalance in the distribution of the organelles to dendrites and axon terminals.

Altered mitochondrial homeostasis, involving mitochondrial dynamics, movement, biogenesis, clearance, and overall function, is prone to dysregulation in ALS. It can cause an avalanche of pathological changes in neurons and other ALS-affected cells, since mitochondria actually serve as hubs for cellular metabolism that are involved in the synthesis of essential molecules, ATP production via oxidative phosphorylation, excitotoxicity, calcium ion transport, redox homeostasis, axonal transport, apoptotic signaling, and other crucial events, thereby regulating cell fate [13,14,56]. If mitochondrial homeostasis is dysregulated, not only oxidative phosphorylation is diminished, but also, the impaired mitochondrial respiratory chain can generate extraordinarily high levels of ROS. This can further damage mitochondria and lead to the release of mitochondrial proapoptotic proteins (cytochrome c, the apoptosis-inducing factor, etc.) and, ultimately, to programmed cell death. Neuronal cells, in particular motor neurons, are especially vulnerable to mitochondrial malfunction due to their high energy demands and inability to switch to glycolysis when oxidative phosphorylation in the organelles is disrupted. Furthermore, neurons are characterized by unusually long processes, and they are more dependent on mitochondrial trafficking than any other cell type [14,16]. In addition, neurons are terminally differentiated, highly long-lived post-mitotic cells that cannot transfer old and damaged organelles to daughter cells by cell division. Therefore, sufficient quantity, proper distribution, and function of the organelles are required for neuronal survival, with ATP supply correlated with synaptic integrity [14,16,17].

The last few years of research in the field of genetics and the use of a number of in vitro and in vivo models of ALS have greatly advanced our understanding of the mechanisms of disease-associated changes in neuronal mitochondrial homeostasis and stress response. The most commonly studied ALS-linked mutations are *SOD1* point mutations and variants, GGGGCC24+ hexanucleotide repeat expansion (HRE) in the *C9ORF72* gene, *TARDBP* point mutations and variants, and mutations in the *FUS* and *TBK1* genes. The main disease-causing mutations responsible for disturbances in mitochondrial homeostasis in neurons in genetic in vivo and in vitro models of the pathology are summarized in Table 1.

**Table 1:** The main ALS-causing mutations responsible for disturbances in mitostasis in neuronal and other cells in genetic in vitro and in vivo models of the pathology.

Disease-Causing Mutations	Most Common Disturbances in Mitochondrial Homeostasis	Encoding Proteins and Their Function	Commonly Used In Vivo or In Vitro Models	Ref.
*In vivo* Models				
*SOD1* point mutations and variants	Disruption of the cristae and disintegration of the mitochondrial structure; Vacuolar degeneration; Dysfunction of OXYPHOS; Mitochondrial Ca^2+^ uptake disorders; Mitochondria distribution impairment; Reduced mitochondrial density	Cu/Zn superoxide dismutase 1 (SOD1) protein catalyzes the dismutation of the superoxide radical into hydrogen peroxide and molecular oxygen; it plays a role in the cellular homeostasis of ROS.	Cu/Zn superoxide dismutase 1 (SOD1)-G93A mouse models	[23,27,57,58,59,60,61]
			G93R-mSOD1 zebrafish model	[8,59,60,61]
A GGGGCC24+ hexanucleotide repeat expansion (HRE) in the *C9ORF72* gene	Defective mitochondrial bioenergetic function; Mitochondrial fragmentation; Reduced protein level of the ATP synthase complex	The function of C9ORF72 remains unknown, but it has been suggested that it plays a role in protein trafficking	FVB-C9orf72 mouse model;Rodent models of C9orf72;C9orf72 knockdown zebrafish model	[62,63]
*TARDBP* point mutations and variants	Abnormal mitochondrial morphology and motility	TAR DNA-binding protein 43 (TDP-43) protein participates in transcriptional regulation through RNA/DNA and protein–protein interactions, RNA processing and splicing regulation	TDP43-Q331K mouse model; TDP-43G298S mice (over 20 mouse models of TDP-43); TDP43-A315T zebrafish model	[64,65,66,67]
*FUS* and *TBK1* variants	Mitochondrial fragmentation; Damaged mitochondrial cristae; Hyperfused and elongated mitochondria	Fused in sarcoma (FUS) protein is a DNA/RNA-binding protein that participates in in DNA damage, mRNA splicing, transport, transcription, and translation.	Drosophila models of FUS-related ALS; hFUSWT mice; FUS(1-359) mice	[68,69,70,71,72,73]
			Tbk1fl/flNestin-Cre mice	[74,75,76]
***In vitro* models**				
Mutations in *SOD1*, *FUS*, *TARDBP*, *C9orf72*, *SFPQ*, and others	Disorders in mitochondrial morphology and movement; Impairment of mitochondrial calcium buffering; Disruption of endoplasmic reticulum (ER)-mitochondria tethering and signaling		Human induced pluripotent stem cells (hiPSCs)-derived motor neurons, astrocytes, and microglia from patients with *SOD1*, *FUS*, *TARDBP*, *C9orf72*, and other mutations	[77,78,79,80,81]
Mutations in *SOD1*, *FUS*, *KIF5A*, *PFN1*, and others	Altered mitochondrial shape and size; Reduced mitochondrial movement; Matrix swelling and other structural defects in mitochondria; Decreased mitochondrial respiration; Decrease in the number of mitochondria.		Cultured murine primary neurons co-transfected with mutant fALS-related proteins; human cells expressing mutant derivatives	[82,83,84,85]

Thus, a number of ALS-associated genes, including the very first identified (*SOD1*) and recently discovered genes (*FUS*, *TBK1*, *TARDBP*, *C9orf72*, *KIF5A*, *OPTN*, *SFPQ*, and *SQSTM1*), have been associated with structural and functional disorders in mitochondria and mislocalized mitochondrial networks. Some mutant proteins associated with ALS can directly interact with mitochondria. For example, the deposition of the misfolded SOD1 protein within mitochondria and in close proximity to the mitochondrial surface can interfere with the activity of the respiratory chain complexes, leading to structural abnormalities and defects in oxidative phosphorylation [22,23,58]. Mutant TDP-43 forms have a defective nuclear localization signal and can accumulate in mitochondria [67]. The entry of TDP-43 into mitochondria was found to be driven by internal protein motifs that were rich in hydrophobic amino acids, while the deletion of the motifs prevented the mitochondrial import of TDP-43 [67]. Notably, the protein (encoded by *SQSTM1*) and OPTN (encoded by *OPTN*) after their phosphorylation with TBK1 (encoded by *TBK1*) can be recruited into damaged mitochondria to further direct them to autophagosomes, while ALS-linked mutations in these genes may block correct autophagosome formation [25]. Mutations in *PFN1* (profilin 1), identified in rare family cases of ALS, are associated with a deregulated RAB9-mediated autophagy pathway mediating mitochondrial clearance, which leads to a decrease in the number of mitochondria and ATP production in the cells of ALS patients and transfected cell lines [84]. Thus, the dysregulation of mitochondrial homeostasis appears to be shared by several genes related to ALS and may play a critical role in the propagation of motoneuron injury and pathology progression via different molecular pathways.

Most of the mitochondrial research for fALS has been conducted using mouse models. To date, several lines of transgenic mice have been created, differing in the rate of progression of the pathology. The latter correlates with the promoter architecture associated with transcriptional regulation and the number of copies of the mutant gene expressed in transgenic animals [9]. In addition, zebrafish and Drosophila models have been used to study the pathology of SOD1-, C9orf72-, FUS- and TDP43-related ALS in in vivo experiments [8,9].

In recent years, significant progress has been made in research into the molecular mechanisms responsible for alterations in mitochondrial ultrastructure and function using human induced pluripotent stem cells, (hiPSCs)-derived motor neurons, astrocytes, and microglia from patients with *SOD1*, *FUS*, *TARDBP*, *C9orf72*, and other mutations [77,78,79,80,81]. Furthermore, cultured neurons co-transfected with mutant fALS-related proteins are used to assign functions to the genes and investigate cellular abnormalities, including mitochondrial dysfunction in in vitro studies [73,82,83].

To mimic the sporadic form of the pathology, some chemical (environmental) models of ALS have been created, namely bisphenol A (BPA)-induced motor neuron degeneration in zebrafish and β-sitosterol-β-d-glucoside (BSSG) exposure in mice [86,87]. However, mitochondrial defects in these models have not yet been described.

Intensive studies of tissue samples from patients with sALS revealed many signs of mitochondrial malfunction and dyshomeostasis, including impaired distribution of mitochondria and accumulation of dysmorphic and enlarged organelles, many with deficiencies of essential enzymes, in anterior horn cells, intramuscular nerves, and skeletal muscles [88,89,90,91].

Taken together, genetic studies on animal models, cell culture systems, and patient tissues indicate that the pathogenesis of ALS is closely related to the mechanisms responsible for altered mitochondrial homeostasis, and analysis of dynamic changes in mitochondria may be important for predicting the temporal course of neuron integrity disorders with the progression of the pathology.

## 4. Molecular Mechanisms Responsible for Altered Mitostasis in ALS

**Figure 2:**
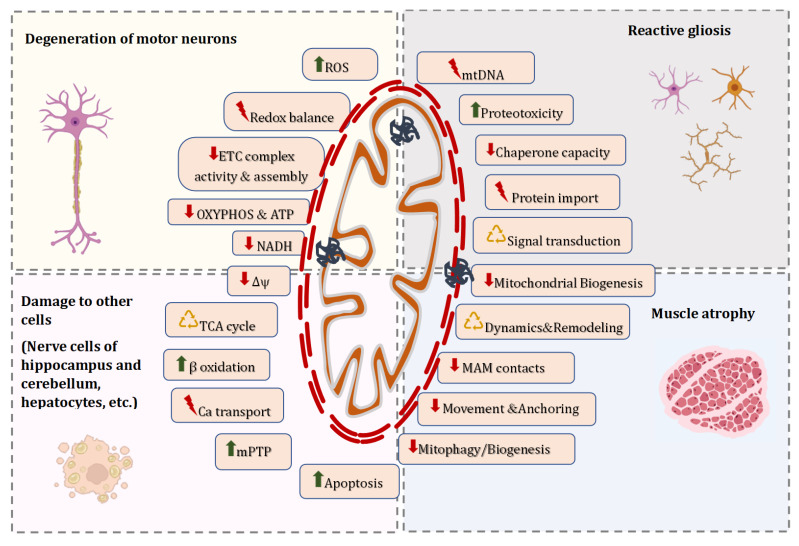
Molecular mechanisms responsible for altered mitostasis in ALS-affected cells, leading to degeneration of motor neurons, reactive gliosis, muscle atrophy, and damage to other peripheral tissues. The misfolding, aggregation, and increasing accumulation of toxic proteins may contribute to their transfer to the mitochondria, which makes their dysfunction one of the early pathological signs of ALS. Mutant SOD1, C9orf72, FUS, TDP-43, and other proteins can be located on the cytoplasmic surface or within mitochondria. Characteristics of mitochondrial dyshomeostasis are changes in the number and distribution of mitochondria in cells; deep ultrastructural abnormalities of organelles; impaired mitochondrial dynamics, biogenesis, axonal transport and mitophagy; de-creased activity of mitochondrial electron transport chain (ETC) complexes and tricarboxylic acid (TCA) cycle enzymes; decreased ATP synthesis and NAD(P)H level; the decreased threshold of the mPTP opening; excessive ROS formation; etc. Abnormalities in the mitochondrial network can finally result in bioenergetic crisis and the initiation of cell death signals.

The morphology and dynamics of mitochondrial network, quality control mechanisms, motility, and overall mitochondrial function are closely interrelated pathways that play a fundamental role in the dyshomeostasis of mitochondria in neurons, surrounding glial cells, myocytes, and many other cell types across peripheral tissues. The studies of fALS and sALS strongly suggest that these processes are affected in the course of the disease. Figure 2 summarizes the key mechanisms that have been proposed to contribute to alterations in mitochondrial homeostasis in ALS-affected cells, leading to degeneration of motor neurons, reactive gliosis, muscle atrophy, and damage to other peripheral tissues.

Accumulating evidence indicates that pathological changes in mitochondria manifest themselves long before the appearance of the first symptoms both in animal models and ALS patients and correlate with denervation. The development of mitochondrial dysfunction was found to be accompanied by a decrease in the availability of ATP for axonal transport and immobilization of damaged or depolarized mitochondria, which facilitates their removal via different autophagy degradation pathways [19,92]. Disorders in homeostasis of cellular calcium due to glutamate excitotoxicity can be associated with dysfunction of mitochondria as the main buffer system for this ion [93]. “Defective” mitochondria can release proapoptotic proteins, including cytochrome *c*, the apoptosis-inducing factor, and others, which cause neuronal death [94]; an increase in the level of ROS due to impaired functioning of mitochondrial respiratory chain complexes can lead to oxidative stress and neuroinflammation [95].

Molecular mechanisms responsible for altered mitostasis in ALS-affected cells contribute to excitotoxicity, oxidative stress, energy deficiency and, ultimately, the death of motoneurons and other cells. Damaged mitochondria can release a number of proapoptotic factors and inflammatory response activators called damage-related molecular patterns, which creates a cycle of direct communication between mitochondrial disorders, inflammation, and cell degeneration [4,10,30,31,32,33,34]. Further comprehensive analysis of molecular mechanisms of mitochondrial dyshomeostasis may elucidate the multifactorial pathogenesis of ALS.

### 4.1. Alterations in Morphology of Individual Mitochondria and Dynamics of the Mitochondrial Network in ALS

A growing body of evidence suggests that abnormalities in the morphology of individual mitochondria and impaired dynamics of the mitochondrial network could be causally involved in damage to motor neurons and other cells in ALS genetic animal models, transfected cells, and patients with both sporadic and familial forms of the disease. For example, ultrastructural rearrangements and the associated decrease in the enzymatic activity of respiratory complexes of mitochondria have been described in detail in the cells of patients with ALS associated with mutations in SOD1 and SOD1-transgenic animals [91,96,97,98,99]. In the motor neurons of SOD1 mice, the structural signs of mitochondrial damage involve pathological changes in the size, quantity, shape, matrix volume, the organization of mitochondrial cristae, and distribution of the organelles, which worsened as the pathology progressed. It was found that vacuolized mitochondria appear already at the early stages of motor neuron degeneration in SOD1 mice, with mutant SOD1 contributing to the elongation of the outer mitochondrial membrane and the expansion of the intermembrane space [100]. Some studies showed that mitochondria in motor neurons can cluster and become spherical [101], which disrupts the anterograde transport of mitochondria by reducing the content of mitochondrial Rho GTPase 1 (MIRO1), a protein necessary for the transport of mitochondria from the axon to the neuron body [102]. Elongated mitochondria in dendrites and structural defects of the inner mitochondrial membrane were detected in the motor neurons of TDP-43A315T transgenic mice [103]. ALS-associated protein FUS has been found to interact with the mitochondrial proteins heat shock proteins 60 (HSP60), also known as chaperonins, β-subunit of ATP synthase, and respiratory chain complex mRNAs; mutations in FUS, which induce FUS redistribution to the cytoplasm, have been associated with mitochondrial fragmentation and network disruption in transfected cells and patient tissues [71,85]. There is accumulating evidence in both mouse and Drosophila models that mitochondrial morphology abnormalities associated with motor neuron degeneration are a very early pre-symptomatic manifestation of the disease [68,100,103].

Similar destructive and degradative rearrangements of mitochondria have been reported in motor neurons and other cells of ALS patients. Thus, ultrastructurally abnormal mitochondria (including giant mitochondria) were found in the spinal cord, brain, skeletal muscles, and some other ALS-affected tissues [30,104,105]. Electron microscopy data often revealed a disturbance in the packaging of mitochondrial cristae and the appearance of procrystalline inclusions inside the organelles. Naumann and co-authors described mitochondrial shortening, damage to mitochondrial cristae, fragmentation and severe disruption of the mitochondrial network along with subsequent neurotoxicity in cells of FUS-ALS patients [106]. Teyssou et al. reported that in patient cells (lymphoblasts) or tissues (post-mortem) carrying *PFN1* mutations, mitochondria were sparse, swollen, and enlarged [84]. Additional signs of degenerative changes in the mitochondria were an expanded electron-transparent mitochondrial matrix with a disrupted architecture of the cristae membrane, the presence of various ingested cytosolic components, and the occurrence of membranous vesicles in close contact with the outer mitochondrial membrane [84].

Aggravating changes in the structure of mitochondria and the mitochondrial network upon ALS progression suggest an imbalance of the processes of mitochondrial dynamics, a key mechanism that regulates the maintenance of a dynamic continuum between fragmented and fused states of organelles. Mitochondrial dynamics involves coordinated cycles of the fusion and fission processes and is essential for cells to acquire the mitochondrial morphology required for ever-changing metabolic needs, enabling cells to respond adaptively to stressful stimuli [54]. A potential consequence of the disruption of these processes is the inability to provide cells with functional mitochondria.

Fission/fusion dynamics is mediated by interactions between conserved members of the dynamin superfamily of high-molecular-weight guanosine triphosphatases (GTPases). Dynamin-related protein 1 (DRP1), fission factor 1 (FIS1), and mitochondrial fission factor (MFF) participate in mitochondrial division [54]. It is known that DRP1 compresses mitochondria, gathering and forming a ring structure at the sites of constriction; FIS1 is anchored in the outer mitochondrial membrane and participates in the recruitment of DRP1 using its cytosolic domain. It is believed that in addition to these proteins, the mitochondrial dynamic proteins MiD49 and MiD51 may participate in the fission processes [107]. In general, mitochondrial fission is necessary to provide sufficient numbers of mitochondria, sustain cell polarity, and aid in eliminating damaged mitochondria. In addition, sumoylation of Drp1 is required to maintain the endoplasmic reticulum-mitochondrial signaling network necessary for the initiation of apoptotic cell death [108]. In contrast, mitochondrial fusion allows the internal components of the organelles to mix, ensuring a more structurally and functionally homogenous mitochondrial network. The fusion of the outer mitochondrial membranes is mediated by the proteins mitofusins 1 and 2 (MFN1 and MFN2), while optical atrophy protein 1 (OPA1) is responsible for the fusion of the inner membranes [54]. OPA1 is known to also be involved in maintaining the structure of cristae membranes in mitochondria [109].

The data on the fission/fusion dynamics in neuronal mitochondria throughout the course of ALS are contradictory. Some studies showed that in association with ultrastructural changes in mitochondria, the level of proteins responsible for both mitochondrial fusion (MFN1, OPA1) and fission (DRP1 and FIS1) increases in the spinal cord of SOD1-transgenic mice before the onset of motor symptoms [110,111]. In this context, Joshi and colleagues reported excessive Drp1-mediated mitochondrial fragmentation both in fibroblasts derived from patients with multiple forms of fALS and in SOD1-mutated motor neurons [112]. An imbalance between fission and fusion was observed in human fibroblasts with *C9orf72* mutation due to elevated MFN1 level [113].

In contrast, at the symptomatic stage of ALS, a decrease in the expression of MFN1 and OPA1 was detected, but not DRP1 and FIS1 [114]. Palomo and co-authors demonstrated that SOD1 mice have a reduced level of MFN2 in the spinal cord [115]. C9orf72 mutations were found to cause an increase in mitochondrial content and fragmentation along with loss of mitochondrial cristae [80,116]. It was revealed that mutant TDP-43 proteins can interact with the mitochondrial chaperone prohibitin 2 (PHB2), voltage-gated anion channel 1 (VDAC1), a mitochondrial degradation receptor, and the MFN2 protein, resulting in TDP-43 attachment to mitochondria and disruption of mitochondrial dynamics [117]. Moreover, it was also demonstrated that mutant TDP-43 expression can lead to phosphorylation of serine 637 of the DRP1 protein, thereby abolishing mitochondrial fission [117]. Some studies revealed that the mitochondrial localization of mutant FUS proteins can cause an increase in FIS1 and, as a result, an intensification of mitochondrial fragmentation and ROS overproduction, in addition to a decrease in ATP synthesis [71].

Taken together, these studies suggest that unbalanced mitochondrial dynamics may be a common feature of ALS, and this, in turn, may lead to reduced cell survival. Additional studies of the time course of development of mitochondrial dynamics disorders in various tissues are needed in order to determine the ontogenetic features of disease severity and progression.

### 4.2. Functional Signs of Mitochondrial Damage in ALS

Changes in the structure of mitochondria in ALS are interrelated with disorders in the overall function of organelles, which are mediated by a number of molecular pathways (Figure 2) and may exhibit a broad influence on neuronal survival. For example, in the mitochondria of the spinal cord of ALS patients, the enzymatic activity of respiratory complexes I + III, II + III, IV and citrate synthase decreased [18,96]. In parallel, a decrease in the ATP/ADP ratio was reported in the cells of ALS patients, particularly in individuals with the SOD1 and C9orf72 mutations [118]. A similar pattern was also observed in the mitochondria isolated from spinal cord and brain neurons, liver, and skeletal muscle of SOD1-transgenic mice, which led to the disruption of oxidative metabolism and mitochondrial NAD(P)H dynamics [23,30,119]. Noteworthy, a drop in the membrane potential of mitochondria, one of the main indicators of the functional activity of organelles, was observed both in cellar and animal models of ALS [120,121]. Altogether, these data indicate a decrease in the overall number of intact mitochondria in the cell and reduced integrity of the mitochondrial membranes. Many studies indicated overproduction of ROS by the respiratory chain and increased oxidative damage to mitochondria of the spinal cord in model animals and patients with ALS [122,123,124].

Mitochondrial ionic homeostasis, mainly Ca^2+^ transport and signaling, is involved in heterogeneous functions ranging from the control of metabolism and ATP production to the regulation of cell death. Increasing evidence suggests that ALS is associated with the malfunction of mitochondrial Ca^2+^ transport systems, which results in the dysregulation of cytosolic [Ca^2+^] and specific Ca^2+^-dependent events [120]. Studies have revealed that loss-of-function mutations in the neuronal Sigma 1 receptor (SIGMAR1) disturb mitochondria-associated membrane (MAM) contacts, impairing calcium signaling and associated mitochondrial dynamics and retrograde transport, while the SIGMA1-receptor agonist SA4503 restores calcium flux in mitochondria and reduce neuronal cell death in a SOD1-G93A mouse model [125,126].

It was shown that the mitochondria of skeletal muscles of SOD1-transgenic mice have a low ability to accumulate Ca^2+^ ions, which leads to an increase in the concentration of these ions in the cytoplasm. In addition, SOD1-transgenic mice demonstrated increased susceptibility of mitochondria to the induction of the calcium-dependent non-selective permeability transition pore, which is involved in the initiation of apoptosis [93,127]. The mitochondrial permeability transition pore (mPTP) is a multiprotein complex that forms a megachannel across the inner and outer mitochondrial membrane when exposed to various stimuli [128]. It is suggested that the proteins of the inner mitochondrial membrane, ATP synthase and adenine nucleotide translocator, also known as the ADP/ATP translocase (ANT), are potential molecular structures involved in the formation of the megachannel. Currently, the only protein whose participation in the mPTP opening is not in doubt is the regulatory protein cyclophilin D, the target of cyclosporin A, alisporivir, and some other blockers. To date, it is recognized that the mitochondrial pore is one of the most important indicators of mitochondrial dysfunction, and its formation in mitochondria can lead to the disruption of ionic homeostasis, collapse of the mitochondrial membrane potential, massive swelling of the organelles, release of pro-apoptotic proteins, and, eventually, cell death [128].

### 4.3. Modifications in the Mitochondrial Genome in ALS

Although some modifications in mtDNA may represent a risk factor for ALS, the presence of a few mutations in the mitochondrial genome alone is not enough to directly lead to ALS-related neurodegeneration. At the same time, sporadic mtDNA rearrangements and inherited mtDNA point mutations are indirectly associated with ALS [30,129]. In particular, the level of mtDNA, an indirect marker of mitochondrial copy number, was found to decrease in the spinal cord of patients with sALS and fALS [89]. Wang and colleagues demonstrated that FUS mutations are associated with increased damage to DNA in the postmortem motor cortex [130]. In addition, an increased level of oxidative DNA damage was reported in the spinal cord of both sALS and fALS patients [131] and an animal model of ALS [132]. Several clinical investigations described early-onset and rapidly progressive motor neuron diseases that were linked to mtDNA mutations in the cytochrome c oxidase, transfer RNA, and cytochrome b genes [133,134]. It should be noted that nuclear DNA mutations in genes responsible for mitochondrial proteins are also associated with ALS [30].

More recently, a few studies have also evaluated the role of epigenetic modifications, including changes in the expression levels of DNA methyltransferase 3 alpha (*DNMT3A*) and mtDNA methylation, in determining the disease onset [135,136]. It was observed that mtDNA methylation and the levels of DNMT3A increased in the spinal cord and muscles of ALS transgenic mice [137] and the postmortem fractions of neuronal mitochondria of ALS subjects [138]. These findings suggest that the epigenetic modulation of mtDNA may also contribute to the pathogenesis of ALS.

### 4.4. Disorders in Mitochondrial Clearance and Replacement in ALS

Mitochondrial quality control is a key process of mitochondrial homeostasis of cells, aimed at the clearance of damaged mitochondria via mitophagy and their subsequent replacement through mitochondrial biogenesis [16]. Disruption of mitochondrial clearance and replacement was extensively reported in ALS patients and model systems and was suggested to be directly involved in disease pathogenesis.

Mitochondrial biogenesis is a complex process that requires coordination of transcription and replication of the nuclear and mitochondrial genomes. The key regulator providing the transcriptional control of mitochondrial biogenesis is the protein PGC-1a (peroxisome proliferator-activated receptor γ (PPARYY) coactivator-1alfa) [139]. The expression of PGC-1a can be modulated by extracellular and intracellular signals mediated by a number of proteins, including AMPK (AMP-activated protein kinase), CAMK (Ca^2+^/calmodulin-dependent protein kinase), CREB (cAMP response element-binding protein), calcineurin, nitric oxide (NO), and others [139,140,141]. In turn, PGC-1a coactivates several transcription factors, namely nuclear respiratory factor 1 (NRF1), estrogen-related receptors (ERRs), and peroxisome proliferator-activated receptors (PPARs). These transcription factors can trigger the expression of nuclear genes encoding almost all mitochondrial proteins. In addition, mitochondrial transcription factors A and B (TFAM, TFB2M) and mitochondrial DNA polymerase are activated, which stimulates transcription and replication of mtDNA [139,140,142].

Mitochondrial biogenesis is an important stage in the quality control of neuronal mitochondria and a very vulnerable process in neurodegeneration. The data available in the literature on mitochondrial biogenesis in ALS are currently scarce. A decrease in the expression of PGC1a, the main regulator of mitochondrial biogenesis, has been found in the spinal cord and skeletal muscles of patients with ALS, as well as in the spinal cord of SOD1-transgenic mice [143,144,145]. Other investigations demonstrated that the expression levels of proteins responsible for mitochondrial biogenesis (PGC1a, Tfam, ERRa, NRF1, NRF2, and DNA polymerase subunit gamma) did not significantly change in the spinal cord and peripheral blood mononuclear cells from ALS patients [146].

Mitophagy is the process of selective degradation of mitochondria by autophagy. This mechanism is necessary for the cell to maintain the quality of mitochondria by removing the organelles that are subject to protein oxidation and misfolding or exhibit a loss of membrane potential [147]. Thereby, mitophagy protects the cell from apoptosis and mitigates the effects of various environmental factors and toxicants. Mitophagy is stimulated due to the activation of sirtuin-1 (SIRT1) and AMPK proteins. Subsequently, signaling pathways involving PTEN-induced kinase 1 (PINK1) and ubiquitin ligase E3 parkin (PINK1/Parkin pathway) or NIP3-like protein X (NIX/BNIP3) can be activated. When mitochondria are depolarized or injured, these signaling mechanisms are triggered with the formation of autophagosomes and culminate in the complete degradation of damaged mitochondria [55]. Within this scenario, it is the balance between mitophagy and mitochondrial biogenesis that maintains a constant pool of functional mitochondria in the cell.

Mitophagy disturbances have also been associated with ALS, for example, through the involvement of the adaptor protein optineurin, which has been shown to play a role in PINK1-Parkin-mediated mitophagy and can be mutated in familial cases of the disease [148]. Following Parkin recruitment to the outer membrane, optineurin binds to ubiquitinated mitochondria, inducing autophagosome genesis via the ubiquitin-like protein LC3 recruitment. ALS-associated mutations in optineurin impair the clearance of damaged mitochondria [24].

Mutations in additional genes involved in mitophagy, namely *TBK1*, *SQSTMQ1* (encoding for p62), and *VCP* (valosin-containing protein), have been described in fALS [25,76,149,150,151]. For example, *TBK1* loss or mutations have been linked to the inhibition of TBK1-mediated phosphorylation of optineurin, which may result in the delay of mitophagy and accumulation of damaged mitochondria [25,75,76].

The amplification of mitophagy in the spinal cord of persons affected by ALS was confirmed by the finding of an increase in the number of autophagosomes and autophagolysosomes that contain mitochondria [152]. Furthermore, an increase in p62, optineurin, and LC3-II was described in neurons of mutant SOD1 mice [115,153]. Interestingly, a progressive decrease in Parkin level has been reported in both cellular and animal models of ALS [115,154], suggesting that chronic activation of mitophagy leads to Parkin depletion. It was found that in the spinal cord of patients with sALS, the accumulation of the ALS-linked protein TDP-43 is accompanied by a decrease in the cytoplasmic level of the E3 ubiquitin ligase Parkin [154]. In the spinal cord of SOD1-transgenic mice, a decrease in the expression level of PARKIN was observed; however, mitophagy was stimulated. The knockout of the PARKIN protein led to a decline in spinal cord inclusions and an increase in the lifetime of SOD1-G93A model mice [115,155].

Taken together, these data support the suggestion that mitochondrial quality control systems are involved in the pathophysiology of ALS. Importantly, they also demonstrate that unregulated mitochondrial turnover can be a dual-edged weapon, with initial protective effects that can become deleterious in the advanced phase of ALS.

### 4.5. Defects in Mitochondrial Trafficking in ALS

Defects in the trafficking of mitochondria can play an essential role in neuronal cell death, as the distal areas of motor neurons may not be correctly supplied with healthy mitochondria, and damaged mitochondria may not be turned over properly. As is known, damaged or depolarized mitochondria in axons are engulfed by autophagosomes and retrogradely transported to the soma to be degraded after autophagosome-lysosome fusion [147]. The defective retrograde transport of autophagosomes could result in a delay of mitophagy, indicating that mitochondrial trafficking and clearance are interconnected processes [19].

The bidirectional transport of organelles over long distances is mediated via microtubule-based molecular motors, kinesins and dynein, as well as several adaptor proteins that ensure a regulation of mitochondrial movement and distribution. In axons, cytoplasmic dynein is responsible for retrograde transport, while kinesin controls the anterograde transport of neuronal mitochondria. Among adaptor linkers, the best studied proteins are Miro, an atypical Rho GTPase, and Milton, a kinesin heavy chain-binding protein [19]. Milton is linked indirectly with the outer mitochondrial membrane through interaction with Miro, providing the mitochondria-specific axonal transport. Interestingly, the levels of Miro are reduced in ALS models [19,92]. In cultured ALS neurons, Parkin was found to be responsible for Miro degradation and to contribute to the disruption of mitochondrial axonal transport [102], while the overexpression of Miro or ablation of PINK1 prevented defects in mitochondrial trafficking [115].

Ample evidence indicates that a deficiency in mitochondrial axonal transport precedes motor neuron degeneration in ALS [92]. For example, defects in mitochondrial axonal transport and morphology were demonstrated in cellular models of ALS [119,130]. It is important that these anomalies in vivo were also observed in SOD1 and TDP-43 mouse models [66] and Drosophila models of ALS [68,69]. Moreover, FUS mutations were associated with abnormalities in mitochondrial axonal transport in motor neurons and vesicle transport between the endoplasmic reticulum and mitochondria in primary culture models of ALS, iPSC-derived neurons from patients, and transgenic mice [70,81,156].

All the findings are consistent with the “dying-back” hypothesis of ALS [48,49,50], suggesting that mitochondrial axonal transport may contribute to the development of motor neuron degeneration from distant regions and its further progression towards the soma. Indeed, a deficiency in long-range bidirectional mitochondrial transport was predominantly reported at the asymptomatic stage of the disease in SOD1 mutant mice [66], thus indicating that these alterations play an early causal role in ALS neurodegeneration.

## 5. Strategies of Targeted Mitochondrial Therapy for ALS

Currently available drugs for the treatment of ALS have insufficient efficacy and are predominantly compensatory, symptomatic. In clinical practice, only two drugs are approved by the Food and Drug Administration for the treatment of ALS: riluzole (Rilutek, Teglutik), which suppresses excessive motor neuron firing by blocking the release of glutamate, and edaravone (Radicava, Radicut), which acts by neutralizing free radicals. Studies showed that riluzole slightly (by 2–3 months) prolongs the average life expectancy of patients with ALS without affecting its quality [157], while edaravone slows the rate of functional decline at the early stages of disease progression [158].

Difficulties in developing effective genetic therapy are associated with the multifactorial nature of the disease [36]. The identification of novel mechanisms of ALS pathogenesis in recent years has resulted in growing numbers of proposed therapeutic approaches that are used to plan clinical trials. According to ClinicalTrials.gov, over 770 ALS-related clinical trials have been registered to date, but strategies for targeted mitochondrial therapy in ALS are limited. Clinical trials of the combination of dextromethorphan and quinidine (Nuedexta), which affect the demethylation of P450-containing systems in the inner membrane of mitochondria and in the endoplasmic reticulum, have revealed an improvement in the bulbar function (speech, swallowing, and salivation) in patients with ALS (ClinicalTrials.gov identifier NCT01806857). Pridopidine (PL-101), a selective and potent Sigma-1 receptor agonist that affects MAM contacts, is being investigated in the HEALEY ALS Platform Trial (NCT04615923).

Given the fundamental role of dysregulation of mitochondrial homeostasis in disease progression, one can assume that novel ways to pharmacologically or genetically modulate mitochondrial turnover, movement, and dynamics in ALS, as well as restore the bioenergetic balance in the body, may become promising therapeutic strategies. Table 2 summarizes potential pharmacological strategies of targeted mitochondrial therapy for ALS.

### 5.1. Mitochondria-Specific Form of Autophagy as a Potential Target for ALS Treatment

The therapeutic value of modulating the mitochondria-specific form of autophagy in ALS is being intensively studied. Currently, there are no pharmaceutical approaches to selectively regulate mitophagy, but it is possible to target genetically the main components of the mitophagy molecular machinery. For example, the genetic ablation of Parkin in SOD1 transgenic mice decreases mitochondrial protein ubiquitination and restores the levels of Miro and Mfn2, which leads to the normalization of neuronal mitochondrial trafficking and dynamics, a delay in neurodegeneration, and an increase in lifespan [115]. This suggests that Parkin knockout has favorable effects under the conditions of chronic mitochondrial injury. In turn, the overexpression of Miro has been found to rescue mitochondrial axonal transport deficits in a SOD1 cultured neuron model of ALS [102].

Perera and colleagues demonstrated that the knockout of the ATG7 protein gene (protein 7 associated with autophagy), which is involved in autophagy, leads to a decrease in the life expectancy of transgenic mice with the ALS phenotype. Curiously, activation of autophagy with rilmenidine induced severe mitochondrial depletion in motor neurons, and stimulated disease progression and neurodegeneration in treated SOD1 mice [165]. At the same time, recent studies using a transgenic mouse model expressing SOD1 G93A mutant continue to support an ALS-associated impairment of mitochondrial clearance, which can be due to the overexpression of the translocator protein (TSPO, 18 kDa), an outer mitochondrial membrane protein that is widely used as a marker of neuroinflammation [166].

Evans and Holzbaur suggested that therapeutic approaches aimed at enhancing autophagy may not always be useful in ALS [21]. They assumed that subtle strategies such as enhancing the lysosomal function and thus preventing the accumulation of autophagosomes or autolysosomes without sufficient degradation capacity could be more effective. These strategies should be based on a detailed understanding of the cellular mechanisms of autophagy in neurons, taking into account the phase-dependent involvement of autophagy in the progression of familial and sporadic forms of ALS.

### 5.2. PGC-1α as a Drug Target for ALS Treatment

Some studies revealed that a pharmacological activation of mitochondrial biogenesis in ALS may have more successful outcomes. Therefore, the administration of the novel drug VAR10303 (iron chelator/free radical scavenger) to SOD1 mice fed on a high-fat diet induced both the stimulation of mitochondrial biogenesis and an increase in animal life expectancy [159]. The use of butyrate had a similar effect on a cellular model of ALS [160]. Compound R13, a precursor of 7,8-dihydroxyflavone and a selective activator of the TrkB signaling pathway, was also shown to provide an improvement in the survival of transgenic animals expressing G93A-SOD1 [161]. This was accompanied by activation of mitochondrial biogenesis through the AMPK/PGC-1a/Nrf1/Tfam axis, suppression of the development of mitochondrial dysfunction, reduction in motor neuron degeneration and muscle atrophy, and increase in the lifespan of model animals. All this suggests that the signaling pathway involving PGC1α may be one of the main targets in ALS therapy.

### 5.3. Mitochondria-Targeted Antioxidants

Excessive ROS production by impaired mitochondria can trigger chain reactions such as mtDNA mutations, lipid peroxidation, and abnormal protein aggregation. Oxidative damage to electron transport chain complexes can exacerbate ROS generation and eventually lead to oxidative stress and motor neuron injury in ALS [41,42,123,124,131,167]. A number of mitochondria-targeted antioxidants, including MitoQ, cholest-4-en-3-one, oxime (TRO19622), Mito-CP, SS-31, and coenzyme Q10, was also found to slow down a decline in the mitochondrial function in animal and cellular models of ALS [26,27,28,29,30,31]. Of note, coenzyme Q10, an electron carrier of electron transport chain and free-radical-scavenging antioxidant, showed insufficient promise to warrant phase III testing in ALS individuals [163]. Newly synthesized mitochondria-targeted antioxidant compounds with antioxidant activity, such as mitochonic acid 5 (MA-5), have potential as a strategy to combat various pathologies associated with mitochondrial dysfunction [164].

### 5.4. Mitochondrial Biogenetics and Energy Metabolism as Targets of Therapeutic High-Fat Diet in ALS

The state of bioenergetic and oxidative stress associated with a high level of metabolism, dysregulation of mitochondrial oxidative phosphorylation, and increase in the consumption of fatty acids in skeletal muscles are well-documented pathophysiological features of ALS [167]. People with ALS are reported to have a lower body mass index. Therefore, therapeutic targeting of energy metabolism, which contributes to an increase in mitochondrial mass and functional activity in neurons, surrounding glial cells, and myocytes can be one of the most promising strategies for the treatment of ALS.

A growing body of evidence suggests the potential benefits of a high-fat and low-carbohydrate (ketogenic) diet, which leads to moderate weight gain, maintenance of energy status, and increase in life expectancy in various ALS models [168,169]. The ketogenic diet has been shown to increase overall body weight and the survival of spinal cord motor neurons in the SOD1-G93A transgenic mouse model. In this case, the accumulation of the main ketone body D-β-hydroxybutyrate that can serve as an alternate energy substrate for neuronal mitochondria was accompanied by the prevention of rotenone-mediated inhibition of respiratory complex I, thus promoting the ATP synthesis in mitochondria [170]. Further investigations showed that caprylic triglyceride (fractioned coconut oil), a substance metabolized into ketone bodies, also restored healthy energy metabolism and improved the motor function in SOD1 mice [162]. Other studies demonstrated that high-fat diet can induce mitochondrial biogenesis through mitochondria-derived ROS activation of calcium/calmodulin-dependent protein kinase (CaMK)-mediated signaling [171], which may have a beneficial effect on mitochondrial content in ALS.

Thus, the use of high-fat, low-carbohydrate diet therapy based on the fundamental knowledge gained about mitochondrial metabolism and homeostasis in ALS can serve as an important complementary method to existing drug approaches for the management of this devastating disease. The promise of this therapeutic approach in ALS is also related to the fact that the currently developed methods of gene therapy and artificial neurogenesis using stem cells often face numerous difficulties and so far, have extremely limited effectiveness.

## 6. Conclusions

Although research in the last decades has revealed a growing number of causative mutations, the precise mechanisms by which different abnormal proteins, when being subject to misfolding and subsequent aggregation, can cause the degeneration of motor neurons and the development of the ALS phenotype continue to remain unclear. Moreover, the involvement of mutant proteins in various metabolic cascades in the cell significantly complicates efforts to categorize various clusters of pathogenesis and to identify molecular pathways that provoke the onset of the pathological process. Accumulating evidence indicates that ALS is a multifactorial disorder. Mitochondria are the central organelles of multiple energy, metabolic, ionic, redox, and signaling reactions and the earliest target in the pathogenesis of ALS. Mitochondrial fusion/fission, biogenesis, mitophagy, and motility are tightly integrated processes that play a key role in the homeostasis of the mitochondrial network. Taken together, recent data suggest that defects in the systems for maintaining the pool of functional individual mitochondria and their network, along with altered proteostasis and ribostasis, contribute to the progression of ALS. The dysregulation of mitochondrial homeostasis contributes to excitotoxicity, oxidative stress, energy deficiency and, ultimately, the death of motor neurons and other cells. Damaged mitochondria can release a number of proapoptotic factors and inflammatory response activators called damage-related molecular patterns, which creates a cycle of direct communication between mitochondrial disorders, inflammation, and cell degeneration.

Overall, the genetics of ALS strongly emphasizes the relevance of mitochondrial homeostasis dysregulation in disease pathogenesis, which may have potentially beneficial therapeutic implications. Nowadays, the concept of the “magic shotgun, that is, the model “one disease—multiple targets”, has been increasingly used in the development of potential drugs for the treatment of diseases with multifactorial pathogenesis [172]. Within the framework of this concept, simultaneous modulation of multiple targets using a well-coordinated pharmacological approach is essential to achieve the therapeutic effect. However, in the context of ALS, it remains unclear what molecular mechanisms determine the transition of mitochondrial responses from adaptive to maladaptive and proapoptotic ones, and whether the dysregulation of mitochondrial homeostasis really contributes to the initiation of the neurodegenerative process. Further studies are clearly necessary in order to achieve a detailed mechanistic understanding of the pathogenic pathways linking mitochondrial dyshomeostasis and motor neuron degeneration, and whether the regulation of these relationships can modify the clinical course of a severe neurodegenerative disease such as ALS. Comprehensive characterization of mitochondrial instability and dyshomeostasis in the cells of affected tissues and their underlying molecular mechanisms could provide insight into novel therapeutic strategies, which may have a significant impact on current symptomatic therapies and personalized treatment programs for patients with sALS and fALS.

## Figures and Tables

**Table 2:** Potential pharmacological strategies for mitochondrial therapy in ALS.

Pharmacological Agent	Molecular Targets	Therapeutic Effect	References
Pridopidine (PL-101)	Sigma-1 receptor agonist, MAM contacts, mitochondrial calcium homeostasis	Enhancement of bulbar and speech function in ALS patients	HEALEY ALS Platform Trial ID NCT04615923
Nuedexta (Dextromethorphan and Quinidine)	Effectors of P450-containing systems in the inner membrane of mitochondria and in the endoplasmic reticulum	Improvement in bulbar function (speech, swallowing, and salivation)	ClinicalTrials.gov ID NCT01806857
VAR10303	Iron chelator, free radical catcher	Stimulation of mitochondrial biogenesis and an increase in animal life expectancy	[159]
Sodium butyrate	Broad-spectrum antioxidant	Stimulation of mitochondrial biogenesis and an increase in animal life expectancy	[160]
Compound R13	Precursor of 7,8-dihydroxyflavone, selective activator of the TrkB signaling pathway	Activation of mitochondrial biogenesis, suppression of the development of mitochondrial dysfunction and an increase in animal life expectancy	[161]
Caprylic triglyceride	Substance metabolized into energy-rich ketone bodies	Restoration of energy metabolism, mitochondrial biogenesis and improvement of motor function in SOD1 mice	[162]
MitoQ	Mitochondria-targeted antioxidant	Improvement in mitochondrial function and neuroprotective effects in animal and cellular models of ALS	[26]
Cholest-4-en-3-one, oxime (TRO19622)	Inhibitor of the components of the mPTP opening: the voltage-dependent anion channel and the translocator protein	Delay in the onset of disease symptoms and increase in animal survival	[27]
Mito-CP	Mitochondria-targeted antioxidant	Improvement in mitochondrial function and neuroprotective effects in animal and cellular models of ALS	[28]
Elamipretide (SS-31)	Mitochondrion-targeted antioxidant	Improvement in mitochondrial dysfunction, synaptic and memory impairment	[29]
Coenzyme Q10	Electron carrier of electron transport chain and free-radical-scavenging antioxidant	Slowdown in the decline of mitochondrial function in animal and cellular models of ALS	[163]
Mitochonic Acid 5	Inductor of ATP synthase dimer formation	Increase in local ATP production and cell survival in various mitochondrial diseases	[164]

## Data Availability

No new data were created or analyzed in this study. Data sharing is not applicable to this article.
